# Effects of storage and toothbrush simulation on Martens hardness of CAD/CAM, hand-cast, thermoforming, and 3D-printed splint materials

**DOI:** 10.1007/s00784-023-05378-4

**Published:** 2023-11-13

**Authors:** Martin Rosentritt, Verena Hickl, Angelika Rauch, Michael Schmidt

**Affiliations:** Department of Prosthetic Dentistry, UKR University Hospital Regensburg, 93042 Regensburg, Germany

**Keywords:** Hardness, Martens, Elastic behavior, Viscoelastic parameters, Creep

## Abstract

**Objectives:**

To investigate Martens hardness parameters of splint materials after storage in liquids and toothbrush simulation.

**Materials and methods:**

Ten specimens per material and group were fabricated (hand-cast CAST, thermoformed TF, CAD/CAM-milled CAM, 3D-printed PS, PL, PK, PV), stored in air, water, coffee, red wine, and cleaning tablets and investigated after fabrication, 24 h, 2- and 4-week storage or toothbrushing. Martens hardness (HM), indentation hardness (*H*_IT_), indentation modulus (*E*_IT_), the elastic part of indentation work (*η*_IT_), and indentation creep (*C*_IT_) were calculated (ISO 14577-1). Statistics: ANOVA, Bonferroni post hoc test, between-subjects effects, Pearson correlation (*α* = 0.05).

**Results:**

HM varied between 30.8 N/mm^2^ for PS (water 4 weeks) and 164.0 N/mm^2^ for CAM (toothbrush). *H*_IT_ values between 34.9 N/mm^2^ for PS (water 4 weeks) and 238.9 N/mm^2^ for CAM (toothbrush) were found. *E*_IT_ varied between 4.3 kN/mm^2^ for CAM (toothbrush) and 1.8 kN/mm^2^ for PK (water 2 weeks). *η*_IT_ was found to vary between 16.9% for PS (water 4 weeks) and 42.8% for PL (toothbrush). *C*_IT_ varied between 2.5% for PL (toothbrush) and 11.4% for PS (water 4 weeks). The highest impact was identified for the material (*p* ≤ 0.001).

**Conclusions:**

Storage and toothbrushing influenced Martens parameters. The properties of splints can be influenced by the choice of materials, based on different elastic and viscoelastic parameters. High HM and *E*_IT_ and low *C*_IT_ might be beneficial for splint applications.

**Clinical relevance:**

Martens parameters HM, *E*_IT_, and *C*_IT_ might help to evaluate clinically relevant splint properties such as hardness, elasticity, and creep.

## Introduction

Temporomandibular disorders (TMD) can be effectively treated with splints [1]. These appliances reduce symptoms including pain and functional limitations [2–4]. Splints can be made on gypsum models either by applying methacrylate in the sprinkle-on technique or by vacuum thermoforming [5]. The digitization of the clinical situation—either directly with an intraoral scanner or the extraoral scan of an impression/plaster model—enables the use of computer-aided design/computer-aided manufacturing (CAD/CAM) [6, 7]. Based on the digitalized clinical situation, the occlusal devices can be designed with a CAD software [8]. CAM manufacturing can then be carried out using subtractive or additive processes. Subtractively machined splints are milled from a prefabricated resin-based blank using a computerized numerical control (CNC) machine [9]. Additive processes include 3D printing with stereolithography (SLA) or digital light processing (DLP) technology [10, 11], in which the splints are built up and cured layer by layer using a liquid photopolymer. Here, the mechanical properties and performance [12] are influenced by both the type of material and the processing. But cleaning and post-polymerization also play an important role in ensuring the material’s properties [13, 14]. In addition to aesthetic [15–17], phonetic, and functional [18] requirements, splints should also meet mechanical requirements such as sufficient and long-term hardness. Stiffness, elastic relaxation, or hardness can influence treatment efficiency and may be associated with iatrogenic implications on the patient’s health [19]. Further on, energy dissipation capabilities and elastic and viscoelastic properties might be advantageous by the utilization of splints.

Hardness is generally defined as the resistance against plastic and permanent deformation measured by methods like Vickers or Brinell hardness testing [20]. Instrumented indentation testing, so called Martens hardness (HM; ISO 14577-1), seems to be a suitable alternative for evaluating the surface hardness combined with information of the elastic and viscoelastic behavior of the splint materials. HM is derived from the applied force (*F*) divided by the indentation surface (As), which is a function of indentation depth (*h*). The constant measurement of force and indentation depth provides a force-indentation depth curve, which allows further interpretation. The indentation modulus (*E*_IT_) is related to the modulus of elasticity [20]. The elastic part of indentation work is expressed by *η*_IT_. The time-dependent response to the indentation of a viscoelastic material [21] can be expressed as the indentation creep (*C*_IT_), showing the relative plastic character of a material, namely, the increase of strain under constant force application. *C*_IT_ might therefore help to estimate the long-term dimensional and mechanical stability of a material [22–24].

The aim of the study was to assess how storage in various liquids (water, coffee, red wine, and denture cleaner solution) and toothbrush simulation affect Martens parameters of different splint materials. As a result of enduring contact with staining solutions and toothbrush simulation, Martens hardness (HM), indentation hardness (*H*_IT_), indentation modulus (*E*_IT_), the elastic part of indentation work (*η*_IT_), and indentation creep (*C*_IT_) are likely to change. The null hypothesis was that these changes would be dependent on the material, fabrication, type of storage, and the duration.

## Materials and methods

**Fig. 1:**
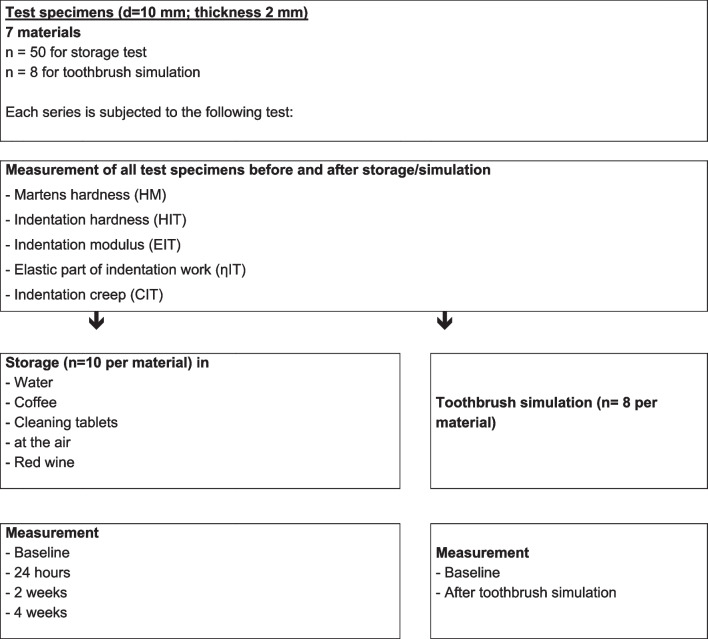
Study design

A total of 58 × 8 (*n* = 10 per material and group) specimens (diameter 10 mm, thickness 2 mm) were hand-cast, thermoformed, CAD/CAM-milled, or 3D-printed (Table 1, Fig. 1). Hand-cast specimens (Palapress vario transparent, Kulzer, Hanau, Germany; mixing ratio 10 g powder, 7 ml liquid) were poured in silicon (VPS Hydro Putty, Henry Schein, Langen, Germany) molds and polymerized in a pressure pot (55°C and 2 bar). Thermoforming of clear foils (Erkodur, 2.00 mm, ∅ 120 mm; Erkodent, Pfalzgrafenweiler, Germany) was performed with Erkoform-3D Motion (Erkodent, Pfalzgrafenweiler, Germany). Specimens were milled from PMMA blanks (Optimill crystal clear; dentona, Dortmund, Germany) with Zenotec select ion (Wieland Dental+Technik, Pforzheim, Germany). A 3D-printing job was created with the slicing software (Netfabb, Autodesk, San Rafael, USA; print direction 90° to the building platform; support structures were used; layer thickness 50 μm). The materials LuxaPrint Ortho Plus (DMG, Hamburg, Germany) and KeySplint Soft (Keystone Industries, Gibbstown, NY, USA) were printed (P30+, Straumann, Cares P series, Basel, Switzerland). Specimens were cleaned (P Wash, Straumann, Cares P series Basel, Switzerland) and polymerized (LED; P Cure, Straumann, Cares P series Basel, Switzerland). The materials V-Print splint and Splint Flex (Voco, Cuxhaven, Germany) were printed (Solflex 650, Voco, Cuxhaven, Germany), manually cleaned (2-min isopropanol bath and ultrasonic), and post-polymerized with xenon light (Otoflash G171: 2000 flashes, 2 min cooling, 2000 flashes; NK Optik, Baierbrunn, Germany). All supports and protrusions were removed with burrs and sandpaper. Polishing was performed with a finishing buff and polishing paste (polishing unit: WP-Ex 2000 II; Wassermann, Hamburg, Germany). Finally, all specimens were cleaned in an ultrasonic bath (35°C, 10 min, Sonorex super RK 102 H, Bandelin electronic, Berlin, Germany).

**Table 1:** Materials, fabrication, and composition

System	Material	Device	LOT	Processing
Thermoforming foil “TF”	Erkodur, 2.00 mm, 120 mm^1^ (*Erkodent, Pfalzgrafenweiler, Germany*)	Erkoform-3D Motion (*Erkodent, Pfalzgrafenweiler, Germany*)	Thermoplastic material: polyethylenterephtalate PET-G	Thermoforming
Cast system MA “CAST”	Palapress vario transparent^2^ (*Kulzer, Hanau, Germany*)	Hand-cast	Methyl methacrylate-copolymer, methyl methacrylate, dimethacrylate	Pressure pot (55°, 2 bar, 15 min)
CAD/CAM “CAM”	Optimill crystal clear^3^ (*dentona, Dortmund, Germany*)	Zenotec select ion (*Wieland Dental+Technik, Pforzheim, Germany*)	Methyl methacrylate, dibenzoylperoxid, methyl 2-methylprop-2-enoat	CAD/CAM milling
Print “PL”	LuxaPrint Ortho Plus^4^ (*DMG, Hamburg, Germany*)	P30+ (*Straumann Cares, Basel, Switzerland*)	Dimethacrylate, EBPADMA, Diphenyl(2,4,6-trimethylbenzoyl)phosphinoxid	*Printing*: direction: 90° to building platform; layer: 50 μm *Cleaning*: P wash (Straumann Cares, Basel, Switzerland), isopropanol *Polymerization*: P cure (Straumann Cares, Basel, Switzerland), LED
Print “PK”	KeySplint Soft^5^ (*Keystone Industries, Gibbstown, NY, USA*)	P30+ (*Straumann Cares, Basel, Switzerland*)	Methacrylate	*Printing*: direction: 90° to building platform; layer: 50 μm *Cleaning*: P wash (Straumann Cares, Basel, Switzerland), isopropanol *Polymerization*: P cure (Straumann Cares, Basel, Switzerland), LED
Print “PV”	V-Print splint^6^ *(Voco, Cuxhaven, Germany)*	Solflex 650 *(Voco, Cuxhaven, Germany)*	Polyester dimethacrylate, BIS-EMA, triethylenglycoldimethacrylat, hydroxypropylmethacrylat, diphenyl(2,4,6-trimethylbenzoyl)phosphinoxid, BHT	*Printing*: direction: 90° to building platform; layer: 50 μm *Cleaning*: ultrasonic (2 min), isopropanol *Polymerization*: Otoflash G171, xenon light: 2 * 2000 flashes
Print “PS”	Splint Flex^7^ (*Voco, Cuxhaven, Germany*)	Solflex 650 (*Voco, Cuxhaven, Germany*)	Dimethacrylate, BIS-EMA, triethylenglycoldimethacrylat (experimental test-material)	*Printing*: direction: 90° to building platform; layer: 50 μm *Cleaning*: ultrasonic (2 min), isopropanol *Polymerization*: Otoflash G171, xenon light: 2 * 2000 flashes

Specimens were stored in water (demineralized water), coffee (Cafet, Netto, Germany—instant coffee mild), red wine (red wine sweet, Vino d’Italia, Italy), and cleaning tablets (Kukident–active plus, Kukident, Germany) in microwell plates. One disc was stored in 1 ml of test liquid. During the immersion procedures, all solvents were exchanged every 4 days. After storage, specimens were rinsed with water and carefully cleaned with a microfiber cloth. Specimens were investigated directly after fabrication (baseline), after 24 h, and 2- and 4-week storage. Toothbrushing was performed with a toothbrush simulator (ZM-3; SD Mechatronik, Feldkirchen-Westerham, Germany; brush: Oral-B 1-2-3 indicator medium (35 mm), Oral B, Germany; slurry: 250 g toothpaste in 1-l demineralized water; load: 250 g, circular 10mm movement, *v* = 40 mm/s, 72,000 cycles) on 8 specimens per material.

Materials were investigated using instrumented indentation testing according to ISO 14577-1. Testing was carried out in a universal hardness-testing machine (ZwickiLine Z2.5, ZwickRoell, Ulm, Germany). The indentation depth was constantly monitored at a loading speed of 0.5 mm/min to a maximum force of *F*max = 10 N, using a Vickers indenter and a dwell-time of 10 s. Unloading was performed at 0.1 mm/min. The recorded force-indentation depth curves were used to calculate Martens hardness (HM), indentation hardness (*H*_IT_), indentation modulus (*E*_IT_), the elastic part of indentation work (*η*_IT_), and indentation creep (*C*_IT_) as defined in ISO 14577-1. Poisson’s ratio of the diamond indenter was set to *ν*_i_ = 0.07 and for the resin-based composite materials to νs = 0.3. Young’s modulus of the indenter was *E*_i_ = 1140 GPa.

Calculations and statistical analysis were performed using SPSS 26.0 for Windows (IBM, Armonk, NY, USA). Homogeneity of the data was controlled with the Shapiro-Wilk test. Means and standard deviations were calculated and analyzed using one-way analysis of variance and the Bonferroni test post hoc analysis. Between-subjects effects were investigated. The level of significance was set to *α* = 0.05. Pearson correlations between the individual parameters were determined.

## Results

**Fig. 2:**
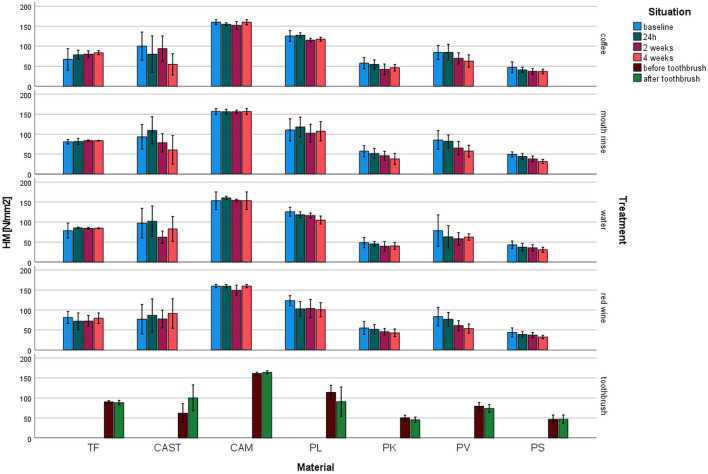
Martens hardness (HM) after different aging/storage treatments and storage times (mean and standard deviation, *significant differences to 24 h measurement, *α* = 0.05)

Martens hardness (HM, Fig. 2): HM varied between 30.8 N/mm^2^ for PS (water 4 weeks) and 164.0 N/mm^2^ for CAM (toothbrush). The highest impact in HM was identified for the material (*p* ≤ 0.001, *η*^2^ = 0.809). TF and CAM show stable values of the different storage conditions and time, whereas CAST and printed systems provided decrease in HM with prolonged storage.

For TF, no influence on HM of any aging was observed (*p* ≥ 0.102). HM of CAM was significantly reduced only after red wine storage (*p* = 0.004, other *p* ≥ 0.165). CAST showed no change of HM (*p* ≥ 0.27) but for the storage in mouse rinse (*p* = 0.038). PL changed HM significantly (*p* ≤ 0.027, other *p* ≥ 0.196) due to storage in coffee, water, and red wine. HM of PK was significantly (*p* ≤ 0.026, other *p* ≥ 0.127) reduced by storage in coffee and mouth rinse. Storage in coffee, mouth rinse, and red wine also influenced PV significantly (*p* ≤ 0.012, other *p* ≥ 0.281. PS changed due to the influence of coffee, mouth rinse, red wine, and water (*p* ≤ 0.043), but not for the toothbrushing (*p* = 0.981).

**Fig. 3:**
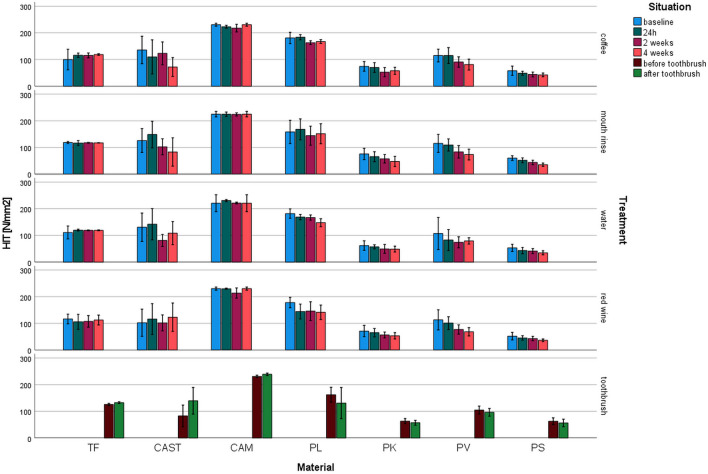
Indentation hardness (*H*_IT_) after different aging/storage treatments and storage times (mean and standard deviation, *significant differences to baseline measurement, *α* = 0.05)

Indentation hardness (*H*_IT_, Fig. 3): *H*_IT_ ranged between 34.9 N/mm^2^ for PS (water 4 weeks) and 238.9 N/mm^2^ for CAM (toothbrush). The highest impact in *H*_IT_ was identified for the material (*p* ≤ 0.001, *η*^2^ = 0.825). TF and CAM showed stable values for the different storage conditions and time, whereas CAST and printed systems showed decreasing HM with prolonged aging.

For TF no influence on *H*_IT_ of any aging was found (*p* ≥ 0.172), but for toothbrushing (*p* = 0.002). *H*_IT_ of CAM was significantly reduced only after coffee or red wine storage and toothbrushing (*p* ≤ 0.003, other *p* ≥ 0.720). CAST showed no change of *H*_IT_ (*p* ≥ 0.050). *H*_IT_ of PL changed significantly (*p* ≤ 0.019, other *p* ≥ 0.263) due to storage in coffee, water, and red wine. *H*_IT_ of PK was significantly (*p* ≤ 0.022, other *p* ≥ 0.074) reduced by storage in coffee and mouth rinse. Storage in coffee, mouth rinse, and red wine significantly influenced PV (*p* ≤ 0.004, other *p* ≥ 0.252), too. PS changed due to the influence of mouth rinse, red wine, and water (*p* ≤ 0.008).

**Fig. 4:**
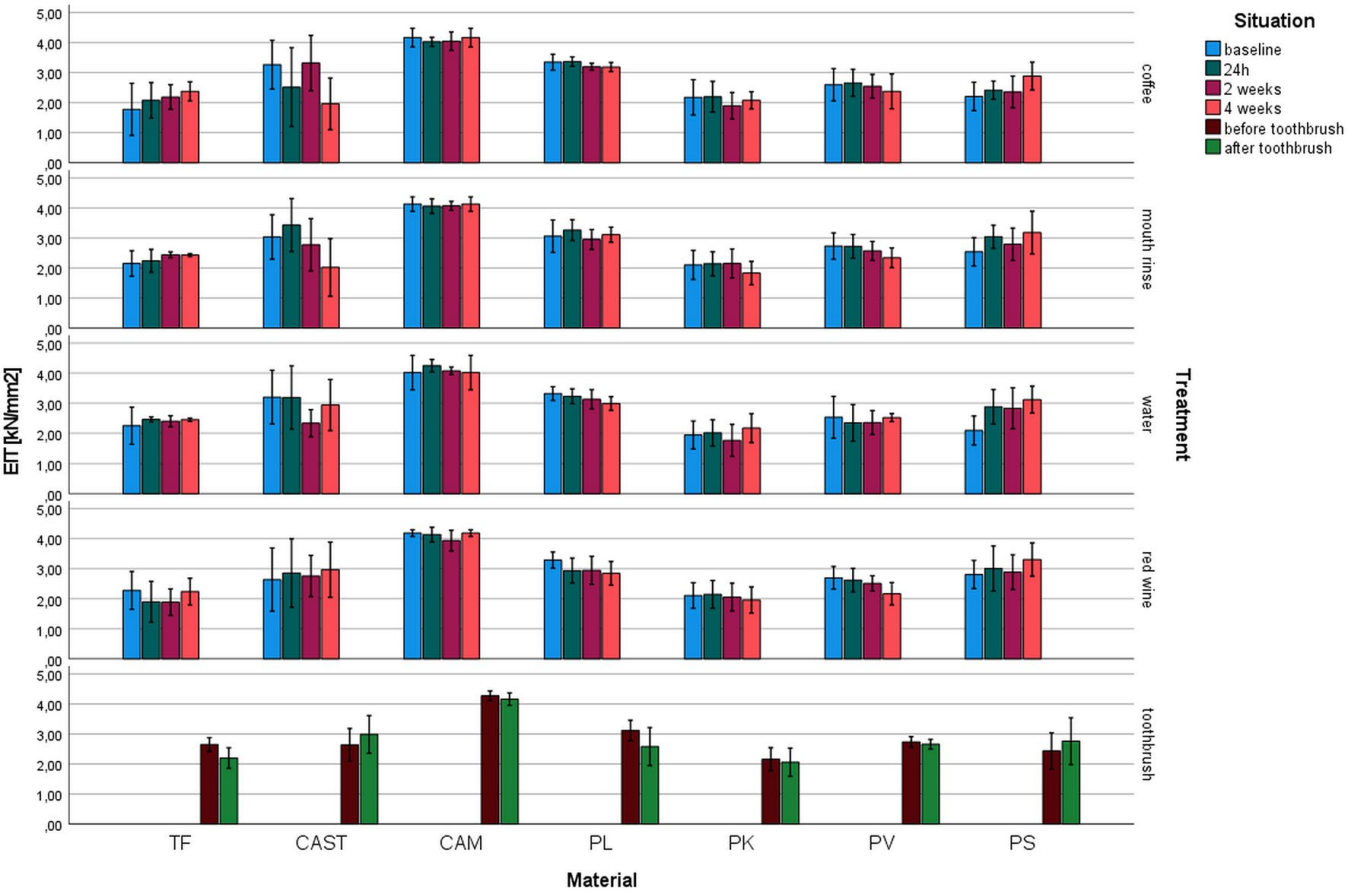
Indentation modulus (*E*_IT_) after different aging/storage treatments and storage times (mean and standard deviation, *significant differences to baseline measurement, *α* = 0.05)

Indentation modulus (*E*_IT_, Fig. 4): *E*_IT_ varied between 4.3 kN/mm^2^ for CAM (toothbrush) and 1.8 kN/mm^2^ for PK (water 2 weeks). The highest impact in *E*_IT_ was identified for the material (*p* ≤ 0.001, *η*^2^ = 0.584). The materials showed only small changes with different storage conditions and time.

For TF, no influence on *E*_IT_ of any aging was observed (*p* ≥ 0.080) but for toothbrushing (*p* = 0.008). *E*_IT_ of CAM was not significantly reduced (*p* ≥ 0.054). CAST showed no changes of *E*_IT_ (*p* ≤ 0.039) due to coffee and mouth rinse storage. *E*_IT_ of PL was not influenced by aging (*p* ≥ 0.053) nor was *E*_IT_ of PK (*p* ≥ 0.308). *E*_IT_ of PV was significantly (*p* = 0.013) influenced by storage in red wine. PS changed due to the influence of coffee and water (*p* ≤ 0.011).

**Fig. 5:**
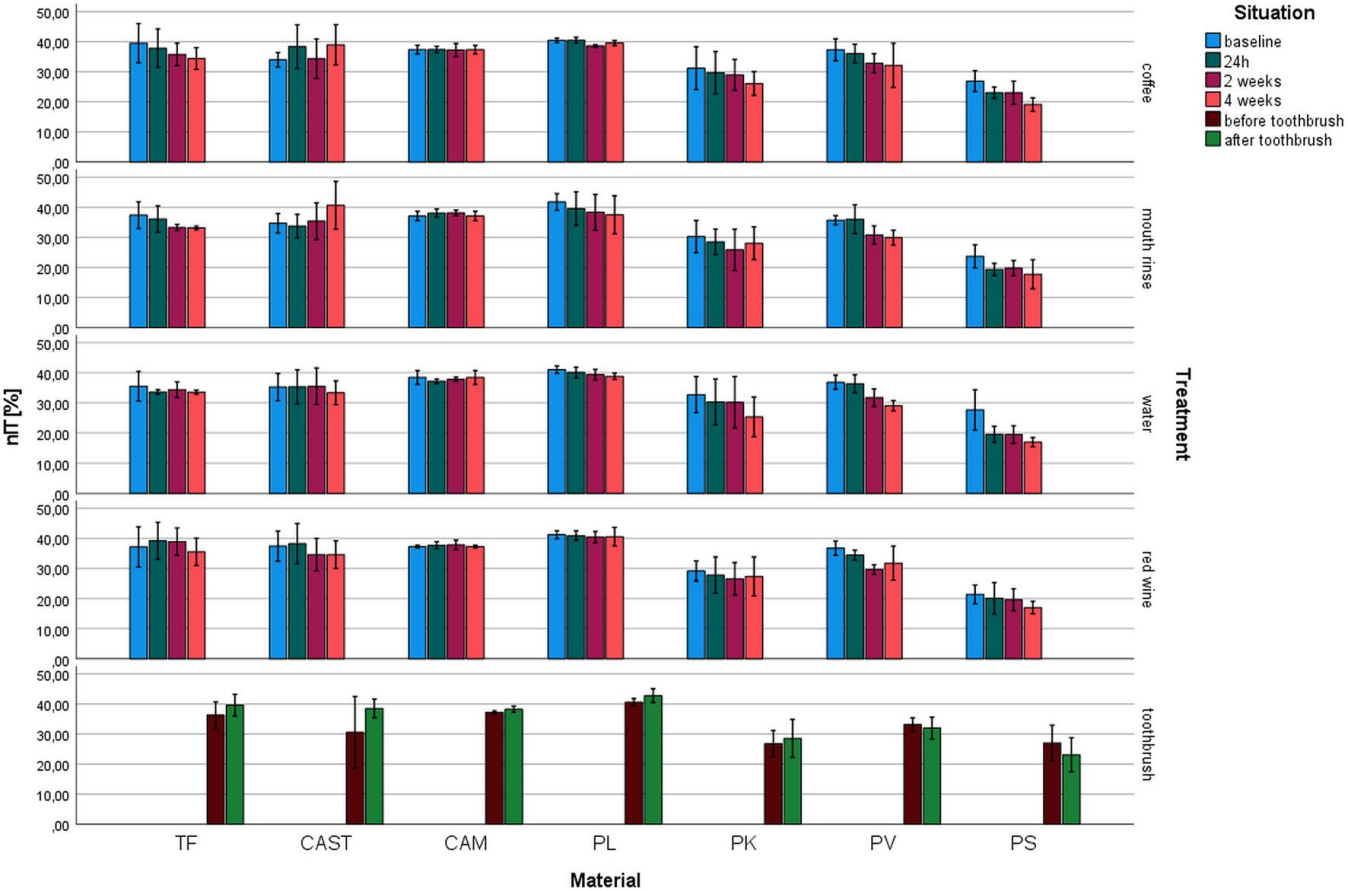
Elastic part of indentation work (*η*_IT_) after different aging/storage treatments and storage times (mean and standard deviation, *significant differences to baseline measurement, *α* = 0.05)

Elastic part of indentation work (*η*_IT_, Fig. 5): *η*_IT_ ranged between 16.9% for PS (water 4 weeks) and 42.8% for PL (toothbrush). The highest impact in *E*_IT_ was detected for the material (*p* ≤ 0.001, *η*^2^ = 0.659). The printed systems showed a decrease in *η*_IT_ with prolonged aging.

For TF, no influence on *η*_IT_ of aging was observed (*p* ≥ 0.123) but for mouth rinse (*p* = 0.010). *η*_IT_ of CAM was not significantly reduced, but for toothbrushing (*p* = 0.022, other *p* ≥ 0.169). CAST showed no changes of *η*_IT_ (*p* ≤ 0.089). *η*_IT_ of PL was not influenced by coffee aging (*p* < 0.001, other *p* ≥ 0.069). PK showed no changes of *η*_IT_ (*p* ≥ 0.160). *η*_IT_ of PV was significantly (*p* ≤ 0.049) influenced by storage, but not by toothbrushing (*p* = 0459). PS changed due to the influence of coffee, mouth rinse, and water (*p* ≤ 0.004).

**Fig. 6:**
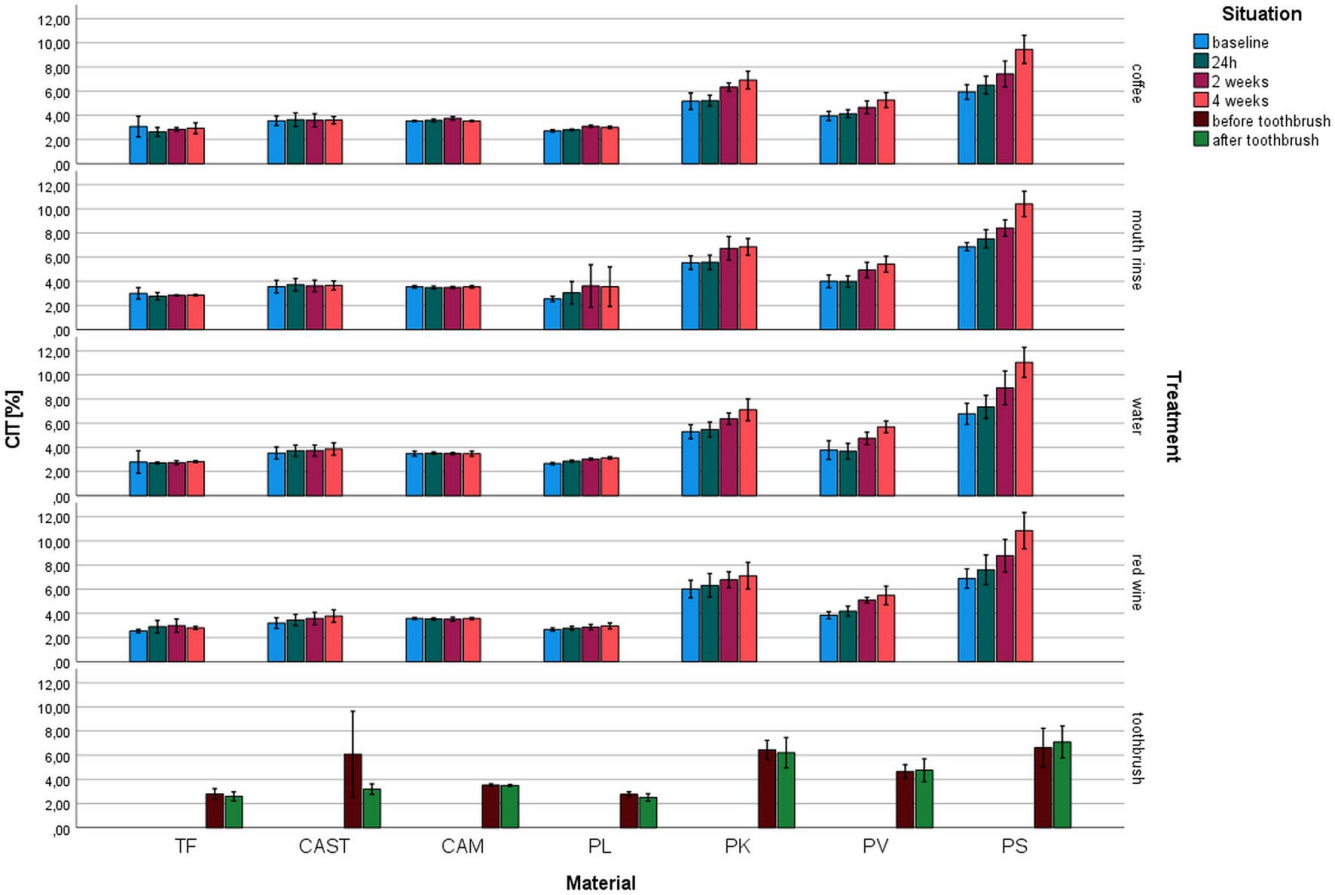
Indentation creep (*C*_IT_) after different aging/storage treatments and storage times (mean and standard deviation, *significant differences to baseline measurement, *α* = 0.05)

Indentation creep (*C*_IT_, Fig. 6): *C*_IT_ ranged between 2.5% for PL (toothbrush) and 11.4% for PS (water 4 weeks). The highest impact in *C*_IT_ was identified for the material (*p* ≤ 0.001, *η*^2^ = 0.869). The values for TF, CAST, and CAM were stable over the storage period, while the printed systems showed an increase with increasing storage time.

For TF, no influence of any aging protocol on *C*_IT_ was identified (*p* ≥ 0.074). *H*_IT_ of CAM was significantly reduced only after coffee exposure (*p* < 0.001, other *p* ≥ 0.387). CAST showed no change of *C*_IT_ (*p* ≥ 0.074) but for toothbrushing (*p* = 0.039). *C*_IT_ of PL changed significantly (*p* ≤ 0.012, other *p* ≥ 0.128) due to storage in coffee, water, and red wine. *C*_IT_ of PK was significantly (*p* < 0.001, other *p* ≥ 0.053) reduced by storage in coffee, water, and mouth rinse. PV and PS were significantly influenced by storage (*p* < 0.001) but not for toothbrushing *p* ≥ 0.530).

## Discussion

The null hypothesis that the Martens parameters are dependent on the material, fabrication, type of storage, and the duration could be partly confirmed.

The examined materials showed clearly different hardness levels ranging between 30 and 165 N/mm^2^. Since Martens hardness is calculated from the course of the indentation depth during loading, it provides information not only about the plastic but also about the elastic material properties. However, e.g., Martens hardness and flexural strength of composite resins are not correlated [25]. To relate the indentation hardness to conventional values, it can also be converted to Vickers hardness, but HV and HM do not necessarily correlate [21]. The HM values identified in the current study were in the order of magnitude that has been measured earlier for other resin-based materials [15, 26, 27]. Varying values have been reported, ranging between ~100 N/mm^2^ [19, 28] for aligner and ~500 N/mm^2^ [27] for resin-based composites, strongly dependent on the type and composition of the materials. General variations might be attributed to different test setups [29]. Differences between composites and resin based-splint materials might be attributed to a different filler content of the materials, which varies between 0% and 87%. The CAD/CAM materials, which can be filled to a higher extent due to the manufacturing technology, showed the highest HM values [30]. Since the printed systems must have a lower viscosity for processing—and are therefore generally filled to a lower extent—their HM hardness is also 20–30% lower [31]. Since the hardness of PL is higher, a higher filler content can be assumed for this material. An additional indication for the higher filler content might be the higher resistance against toothbrush abrasion, because the abrasion is also associated with a loss of resin matrix and filler particles (Valente et al. 2013; Lai et al. 2018).

It is not only the resin composition, but also the conversion, and therefore fabrication and post-processing that might influence hardness. Incomplete polymerization [32, 33] and chemical reactivity could be reasons for the decrease of individual properties [34, 35]. Photo-polymerization influences the structure of dental resin matrices [36], and therefore, the degree of polymer polymerization may be a key to the decrease in HM. It is known that a combined heat- and light-post-curing unit can improve the degree of conversion of 3D-printed occlusal splints [37] and, e.g., the in vitro performance [13, 35]. Light-curing occlusal splint resins have comparable hardness as auto-polymerizing systems (Danesh et al. 2006; Więckiewicz et al. 2014), but the hardness of 3D-printed occlusal splint materials is also influenced by the print angle (Grymak et al. 2021). Against expectations [19, 26, 31], differences between printed materials do not seem to be not or only marginally influenced by manufacturing parameters such as cleaning or post-polymerization. However, the combination of material, processing, and finishing will affect the results. Moreover, the HM results could be influenced by the changed superficial roughness resulting from storage [17].

A decrease of HM was observed during all storage conditions. It was noticeable that only CAD and TF were not affected by storage. The decrease in HM is evident with longer storage time. Similar effects of long-term laydown could also be identified for other properties such as roughness, color, or gloss [17]. This phenomenon will certainly have an impact on the long-term clinical application of the splints. Since the drop in hardness already occurs during water storage and is somewhat equal for all storage media, it can be expected that the materials absorb water. It can be assumed that water absorption reduces the hardness [38, 39] and Martens hardness [26]. A general influence of an individual stirring agent could not be confirmed. Only CAST showed a somewhat indifferent behavior, as with storage in red wine hardness even slightly increased. However, large variations were also observed for this system, which should be attributed to the manual production process and the resulting inhomogeneous structure of the specimens. The results showed that the highest HM stability can be achieved with both milled and printed materials.

The indentation hardness *H*_IT_ is determined using the maximum force and applying tangents to the unloading curve and represents a measure of resistance to permanent and plastic deformation. Since *H*_IT_ shows a similar behavior and ranking as HM, a high proportion of plastic deformation is to be assumed for all materials. The elastic proportion is to be classified as low, especially for parts of the printed materials [40]. The highest stability under clinical bruxism loadings might be expected for CAM and astonishingly one printed system PL. Again, a higher filler content in comparison to the other print systems might be the reason for this behavior [30]. But also storage conditions, such as pH in solution, appear to be related to the hydrophilicity of the matrix and the chemical composition of the filler, which in turn affect sorption and solubility (Örtengren et al. 2001).

The indentation modulus *E*_IT_ is calculated from the indentation relief curve. The increase in the *E*_IT_ values for PS and TF therefore indicates an embrittlement of these materials due to storage. The cast material showed an indifferent picture for *E*_IT_, probably again due to the influence of manual processing. On the other hand, it confirms the same quality of the other production processes: all other materials exhibited a more homogeneous behavior with smaller variations. The combination of material and storage seems to be decisive for the results in *E*_IT_. The elasticity, which is essential for the insertion and removal of the splint during application, therefore seems only minimal. The elasticity of splint specimens is expected to depend on the type of material, their cleaning and post-polymerization. Therefore, *E*_IT_ is also expected to have an influence on the improvement of the dynamic load capacity of splints [12, 13, 27].

A further aspect for the flexibility of the splints is *η*_IT_. The elastic fraction of indentation work (*η*_IT_) is calculated from the areas under loaded and unloaded parts of the load-relief curve. The plastic fraction *W*elast/*W*total 100 % = *η*_IT_ is calculated, which means a high *η*_IT_ is associated with elastic properties. Surprisingly, the examined materials show a comparable *η*_IT_ level. Only two of the printed materials have a lower *η*_IT_ level, i.e., the elastic content is lower. Since the differences between the materials seem to become smaller with *η*_IT_, *E*_IT_ seems to be more meaningful to evaluate elastic properties. Due to the aging process, the elastic portion is reduced in three of the four printed materials, i.e., the materials become visibly brittle. This phenomenon may reduce the retention force of the splint and increase the fracture risk during clinical application [41]. More brittle materials may although provide sharp-edged fractures, which bear the risk for cutting [12].

The *H*_IT_ results correlate with the *C*_IT_ results, where, surprisingly, the investigated materials showed a comparable *C*_IT_ level. *C*_IT_ behaves in the opposite direction to *η*_IT_. Only for two printed materials a higher value could be identified. Since the creep behavior *C*_IT_ describes the further deformation of the material under constant force, the indentation depth for these materials increases under load [40]. Under clinical conditions with continuous load, such as bruxism, the deformations for these materials would therefore be higher. Occlusal deformation might correlate with a loss of contact situation and function. To determine *C*_IT_, the indenter is pressed into the specimen with a constant force over a longer period of time. Polymers with a tendency to creep continuously yield and the penetration depth increases. For three out of four printed materials examined, the value increases due to storage. An influence of the print parameters on the surface quality has been reported [42, 43], and thus, specimens with a lower (e.g., 25 μm) or varying layer thickness (inside 50 μm and outside 25 μm) might show different Martens parameters. All other materials showed a relatively good creep behavior.

Clinically desirable would certainly be a splint material with a high hardness and high resilience, i.e., low creep. Despite the importance of mechanical properties, there is no evidence that the significant differences in in vitro mechanical properties have implications for clinical therapy. For the comprehensive evaluation of the splint materials, the consideration of different Martens parameters seems important, since not all parameters correlate with each other.

## Conclusion

Martens hardness parameters such as hardness (HM), indentation hardness (*H*_IT_), indentation modulus (*E*_IT_), elastic part of indentation work (*η*_IT_), and indentation creep (*C*_IT_) varied significantly between different splint materials.

## Clinical consequence

The clinical behavior of dental splints might be influenced by the selection of materials that feature different elastic and viscoelastic parameters. Materials with high HM, *E*_IT_, and low *C*_IT_ might be beneficial for clinical splint applications.
